# Potential asthma risk factors do not account for global asthma symptom prevalence patterns and time trends in children and adolescents^☆^

**DOI:** 10.1016/j.waojou.2024.100917

**Published:** 2024-06-14

**Authors:** Charlotte E. Rutter, Richard J. Silverwood, Neil Pearce, David P. Strachan, the Global Asthma Network Study Group

**Affiliations:** a Department of Medical Statistics, London School of Hygiene and Tropical Medicine, UK; b Centre for Longitudinal Studies, UCL Social Research Institute, University College London, UK; c Population Health Research Institute, St George's University of London, UK

**Keywords:** Asthma, Wheeze, Prevalence, Time trends, Risk factors

## Abstract

**Background:**

The International Study of Asthma and Allergies in Childhood (ISAAC) and the Global Asthma Network (GAN) conducted a series of global asthma prevalence surveys, between 1990 and 2020, in adolescents aged 13–14 and children aged 6–7 years. We used them to assess whether potential asthma risk factors explain global asthma symptom prevalence trends over this period.

**Method:**

We fitted mixed-effects linear regression models to estimate associations between centre-level risk factor prevalence and both the mid-point asthma symptom prevalence and the change per decade. We also estimated the 2019 asthma symptom prevalence across all included centres.

**Results:**

For adolescents, across 50 centres in 26 countries there was weak evidence that decreasing asthma prevalence over time was associated with regular fast-food consumption and frequent television viewing. However, frequent television viewing, along with heavy truck traffic, were associated with higher prevalence of asthma symptoms at the study mid-point. For children, across 41 centres in 21 countries, no risk factors were associated with time trends in asthma symptom prevalence, but truck traffic and paracetamol in the first year of life were associated with higher mid-point prevalence.

We estimated the 2019 asthma symptom prevalence, across a total of 124 centres, to be 12.8% (11.4%, 14.2%) with little evidence of a difference by age. Low-income countries had lower prevalence (children 5.2% [2.5%, 7.8%], adolescents 5.3% [2.8%, 7.8%]), than lower-middle-, upper-middle- and high-income countries (all approximately 14–15%). Including risk factors in the models did not change the estimates.

**Conclusion:**

Potential asthma risk factors do not seem to explain the global prevalence patterns or time trends. Country income accounts for some of the differences, but the unexplained variation is very high.

## Introduction

Asthma is the most common chronic disease in children^1^ and the prevalence varies dramatically throughout the world.^2^^,^^3^ However, little is understood about its underlying causes and the reasons for global differences in prevalence or the global time trends. Asthma was previously considered to be a “Western” disease, ie, a problem of affluent countries, but there is now a large burden of asthma in many low- and middle-income countries.^4^

Previously published analyses from the International Study of Asthma and Allergies in Childhood (ISAAC) and the Global Asthma Network (GAN) have assessed trends in asthma symptom prevalence over time.^5^^,^^6^ These showed that centres in low-income countries were more likely to have experienced a decrease in prevalence of current asthma symptoms whereas those in lower-middle-income countries experienced increases. Prevalence remained stable on average across centres in upper-middle and high-income countries. However, these analyses were based on a limited number of centres in some of these categories.

Other previous analyses of ISAAC data have identified a number of risk factors that were associated with a higher or lower individual-level risk of experiencing asthma symptoms.^7^, ^8^, ^9^, ^10^, ^11^, ^12^, ^13^, ^14^, ^15^, ^16^, ^17^, ^18^, ^19^, ^20^, ^21^ However, to date there have been no analyses considering the effects of the individual-level risk factors on the centre-level time trends in asthma symptom prevalence. This paper seeks to address whether the centre-level prevalence of previously identified risk factors are related to changes in centre-level asthma prevalence over time. We also estimate the overall prevalence of asthma symptoms across all centres for which time trends data are available.

## Methods

### Study data

GAN Phase 1 was a cross-sectional survey of adolescents (aged 13–14) and children (aged 6–7) that took place between 2016 and 2020. It followed the same protocols as the ISAAC Phases I and III, conducted in 1990–1998 and 2000–2005, respectively. The detailed methods of these studies have been previously reported.^22^, ^23^, ^24^

Information on individual environmental and lifestyle risk factors was collected in ISAAC Phase III and GAN Phase I, but not in the original ISAAC Phase I. Early-life risk factors were included in the children's questionnaire which was completed by a parent but not the adolescent questionnaire which was self-completed. The risk factor data from ISAAC Phase III were used, because this was roughly the mid-point of the studies and included the largest number of centres in which risk factor data were collected. Centres included in this analysis, defined as time trends centres, are those with data from at least 2 phases.

### Variables

Following standard practice in ISAAC and GAN, centre-level prevalence of asthma symptoms for each age-group (the outcome), was defined as the number of individuals answering positively to the question “Have you (has your child) had wheezing or whistling in the chest in the past 12 months?” divided by the total number of respondents in the centre-age-group. Each risk factor prevalence was defined as the number of respondents answering positively to the relevant question (definitions in Supplemental Table S1) divided by the number of respondents with a valid (non-missing) response. (This difference is to remain consistent with other published centre-level results; a missing value to a question on symptoms was taken to mean a negative response given that questionnaires with no answers to any symptom questions were excluded from the original study data. Missing data on symptoms was low (2.0% for adolescents and 2.8% for children). The small amount of misclassification that may occur is likely to only have a very small effect on centre-level prevalence, but it is important that prevalence figures quoted from the studies remain exactly the same regardless which paper they are taken from.)

The risk factors considered in the present analyses include diet, smoking, pets, and medication-related variables. These were selected because they have previously been identified as associated with asthma symptoms in ISAAC.^21^ Each centre's risk factor prevalence was deemed valid, and included in the analyses, if at least 70% of respondents in that age group, within that centre, gave a valid answer for that risk factor question (ie, a response rate of ≥70%). We also included country-level income based on World Bank categories of low-income, lower-middle income, upper-middle-income, or high-income as at 2001 (the study mid-point).^25^ Countries were allocated to 1 of 4 regions of the world, including some combined World Health Organisation (WHO) regions due to data sparsity: Africa and Eastern Mediterranean combined, The Americas, Europe, and South-East Asia, and Western Pacific combined.

Plots and summary statistics of the centre-level prevalence of each risk factor were checked for outliers and assessed for whether the distribution of prevalence values was sufficiently variable to allow discrimination between centres. Those risk factors with an inter-quartile range smaller than 5 percentage-points were removed from further analyses.

The maximum sample included all time trends centre-age-groups with at least 1 valid risk factor prevalence. The common sample included only those centre-age-groups with valid prevalences for all potential risk factors in the analyses.

### Statistical analysis

#### Associations between risk factors and asthma symptom prevalence and time trends

Mixed-effects linear regression models with random intercepts for country and centre, and an outcome of centre-level asthma symptom prevalence, were fitted separately for each risk factor and its interaction with time, adjusting for time as a main effect and for income group and region, along with their separate interactions with time. These minimally adjusted models were fitted on both the maximum sample and the common sample for comparison. Risk factors were scaled to show the effect of a 10 percentage-point change in prevalence as this is a meaningful level of difference between centres. Separate models were fitted for each age group due to the different risk factor information available. Evidence against linearity of the main time effect was checked using a restricted cubic spline.

Fully adjusted models were also fitted on the common sample for each age group, adjusting for all available risk factors and their interactions with time. For comparability with the older age group, additional partially adjusted models were fitted to the younger age group, adjusting separately for all current risk factors and all early-life risk factors. Both fully and partially adjusted models were checked for collinearity between risk factors by comparing the standard errors to those in the minimally adjusted models.

In all the models, time was centred at the start of ISAAC Phase III (Jan 1, 2002) and scaled as the number of decades from this point. This is approximately the time the risk factor data were collected, and also approximately the mid-point of the studies, so the main effect of each risk factor can be interpreted as the effect on the mid-point asthma symptom prevalence. The interaction effect of a risk factor with time is interpreted as the effect on the change in asthma symptom prevalence per decade.

#### Prediction of current prevalence and time trends: income and region-based results

To predict the marginal time trends and current prevalence of asthma symptoms across all time trends centres (overall estimates averaged across the centres in the models, not conditional on covariates), we first extended the income and region-based model from our previous time trends paper^6^ (for all time trends centres regardless of risk factor information, and for both age groups together). The findings are given as estimates of asthma symptom prevalence in 2019, and time trend per decade (over the previous 27 years). Overall estimates, as well as estimates stratified by age group, income group and region are presented. For the income and region-based models we wanted to allow the time trends in each stratum to potentially differ by age group, so a three-way interaction was added. Likelihood ratio tests were used to compare model fit.

The predictive analysis methods were adapted from those previously presented by Cousens et al.^26^ In summary, the regression model was fitted and then the parameter estimates were used to predict the prevalence of asthma symptoms as at GAN Phase I (January 1, 2019) and the change in prevalence per decade across the preceding 27 years. Bootstrapping was used to estimate confidence intervals (CIs) around these estimates, which were summarised at age group, income group and regional levels.

In more detail, after fitting each mixed-effects model, the best linear unbiased predictions (BLUPs) of the random intercepts for country and centre were stored. One observation per centre per age group was retained (i.e., any one time-point, as other covariates are the same), and the dataset was expanded to ensure that there was an observation for each age group for every centre. This formed the basis of the prediction sample. The index time-point was January 1, 2019 and fitted values (from the fixed effects) were predicted and added to the stored BLUPs to create a predicted prevalence per centre per age group. This was repeated with the index time-point January 1, 1992, and then the predicted time trend per decade was estimated from the difference between these two values. The means of these prevalence and time trend predictions were calculated for: i) overall, ii) each age group, iii) by income group/age group, and iv) by region/age group.

This process was bootstrapped 10,000 times to gain (normal approximation) 95% CIs around the mean prevalence and time trends predictions. Resampling was taken at the observation level, ie, a specific time-point for a study centre. Although this is a clustered dataset, resampling was not selected at the centre and country level, as each centre could choose separately to take part in any individual phase. Accounting for clustering in these time trend models is simply to take account of the dependancy between surveys in the same centre, and centres in the same country. The fact that resampling was at the observation level means that any single bootstrap replicate may contain centres with only one time-point. Although centres with only one time-point were removed from the original dataset, since they would not provide any information on time trends, they are not removed here as excluding them from the bootstrapped sample could introduce bias. Additionally, in some bootstrap replicates, centres or countries could be missing entirely, or missing within an age group, therefore without a BLUP or BLUPs. Where a BLUP was missing, it was either copied from another observation of the same centre if available, or for country level BLUPs, from a different centre within the same country. If there were none available, a value was randomly selected from the distribution for the relevant random intercept, ie, a random draw from the normal distribution N (0, σ^2^) where sigma is the standard deviation estimated in the model.

Every bootstrap replicate therefore provided predictions that encompassed the same centres from the prediction sample, one in each age group, even though the underlying regression model was based on a traditional bootstrap sample (with some records excluded and some repeated). This process was run separately for each of two underlying models, one with two-way interactions between time and each stratum and one with three-way interactions between time, age, and income or region. Both models were adjusted for the main effects of age, income group and region.

This prediction method was repeated incorporating the individual level risk factors, based on the previous fully adjusted risk factor models in this paper (separately on each age group due to the different available risk factors) and these were compared to models without risk factors but using the same smaller datasets (with risk factor data available).

This analysis was performed using Stata versions 15 and 17.^27^^,^^28^

## Results

### Descriptive results

**Fig. 1:**
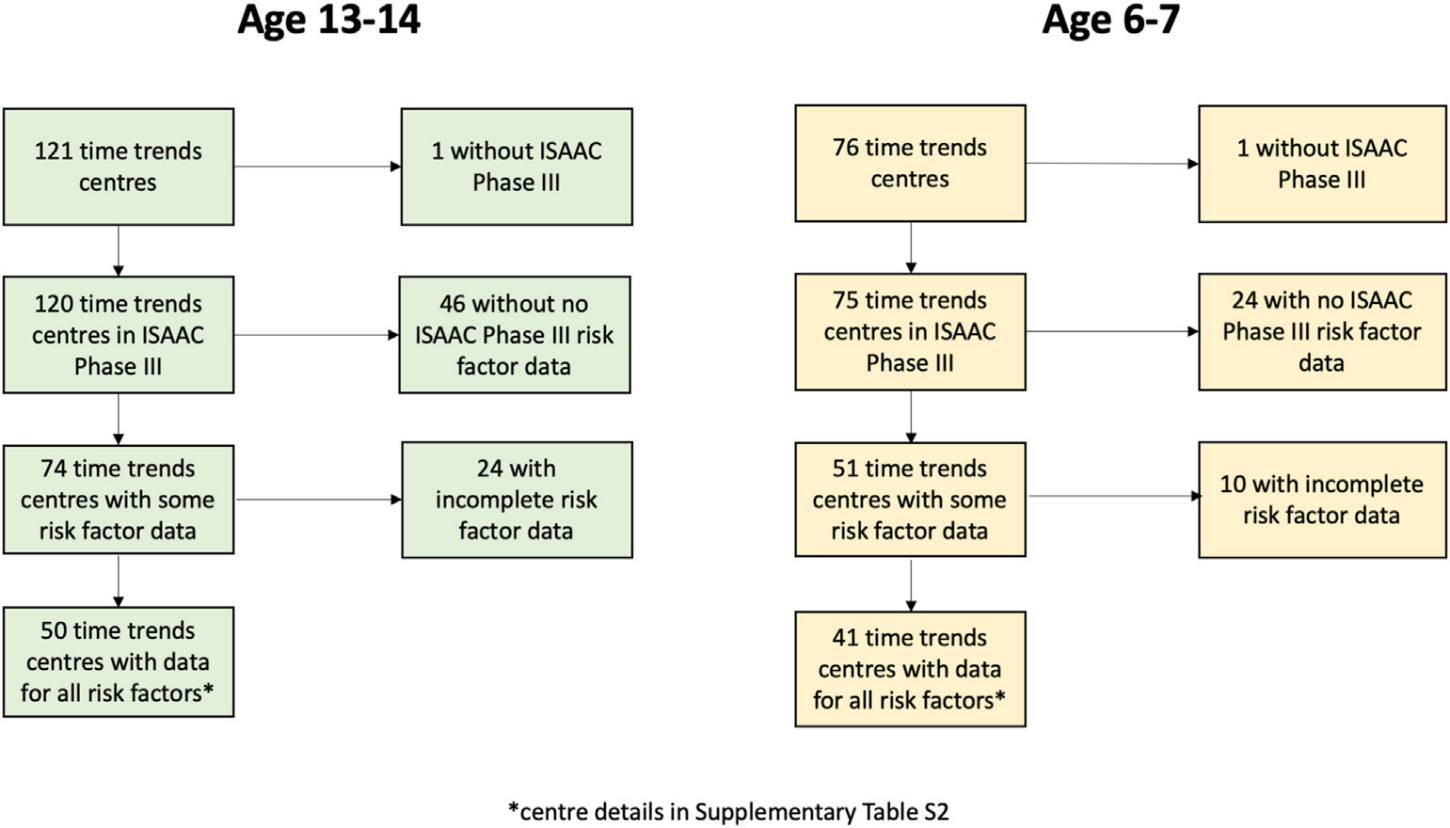
Data flowchart for time trends centres with available risk factor prevalence

**Fig. 2:**
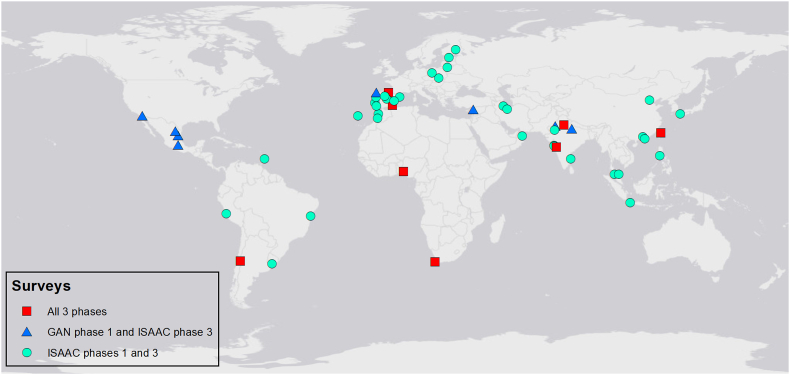
Time trends centres with complete risk factor data for adolescents

**Fig. 3:**
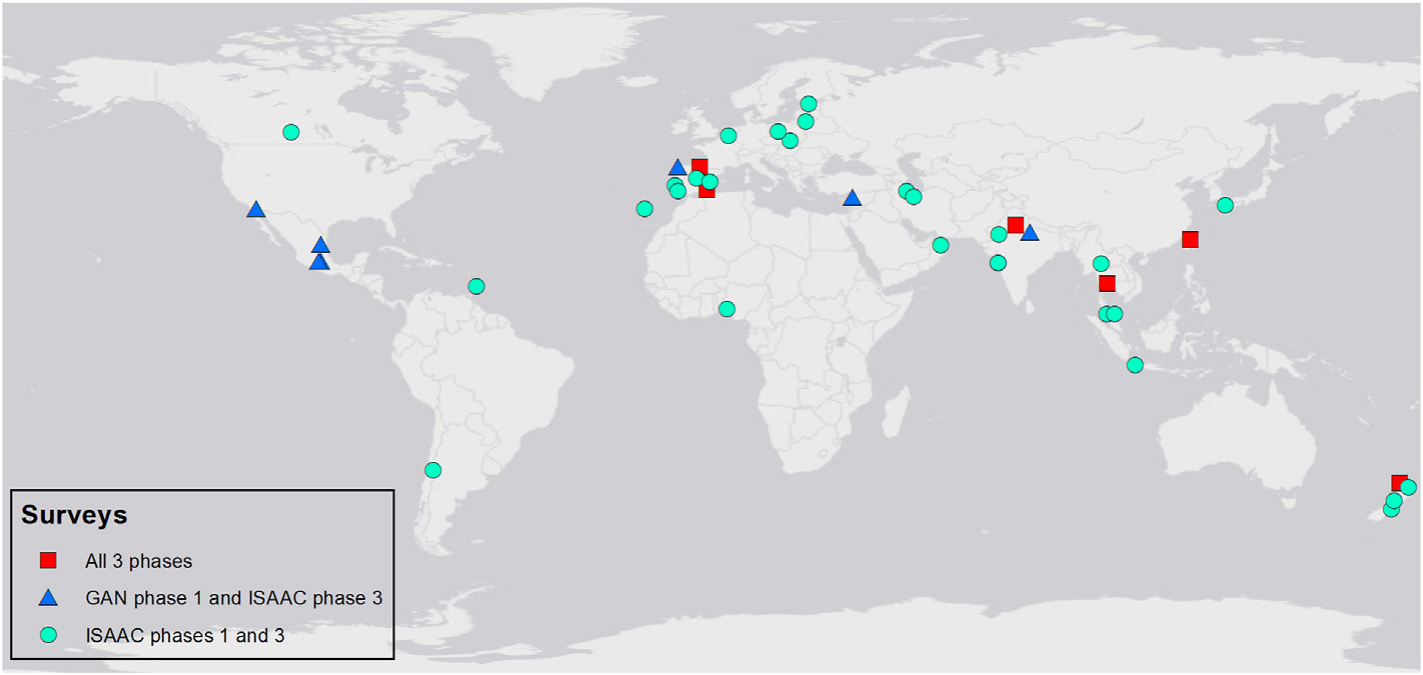
Time trends centres with complete risk factor data for children

Distributions of risk factor prevalence across all available centres were checked; open fire cooking and low birthweight were removed from further analysis as they showed very little variability in prevalence between centres (Supplemental Fig. 1). For adolescents, there were 121 centres for the time trends analyses. Of these, 120 had outcome data from ISAAC Phase III and 74 had valid prevalence (ie, >70% response rate) for at least 1 of the risk factors of interest; 50 centres (across 26 countries) had valid prevalence for all the risk factors (Fig. 1, Fig. 2 and Supplemental Table S2). For children, there were 76 centres for the time-trends analyses. Of these, 75 had outcome data from ISAAC Phase III, 51 had valid prevalence for at least one of the risk factors, and 41 (across 21 countries) had valid prevalence for all the risk factors (Fig. 1, Fig. 3 and Supplemental Table S2). Levels of missing risk factor data in included centres was low, details in Supplemental Table S3.

Distributions of the risk factors in the time trends centres (comparing the maximum sample for that risk factor with the common sample for all risk factors) are shown in Supplemental Fig. S2 for adolescents and in Supplemental Fig. S3 for children. There was sufficient variability across centres and there were no systematic differences between the 2 sets of data.

### Associations between risk factors and asthma symptom prevalence and time trends

The models without individual risk factors showed no evidence against linearity of time trend (p = 0.25 for adolescents and p = 0.38 for children) so remaining models assumed a linear time trend effect. The Level 1 residuals of these models showed no evidence of heteroskedasticity.

**Fig. 4:**
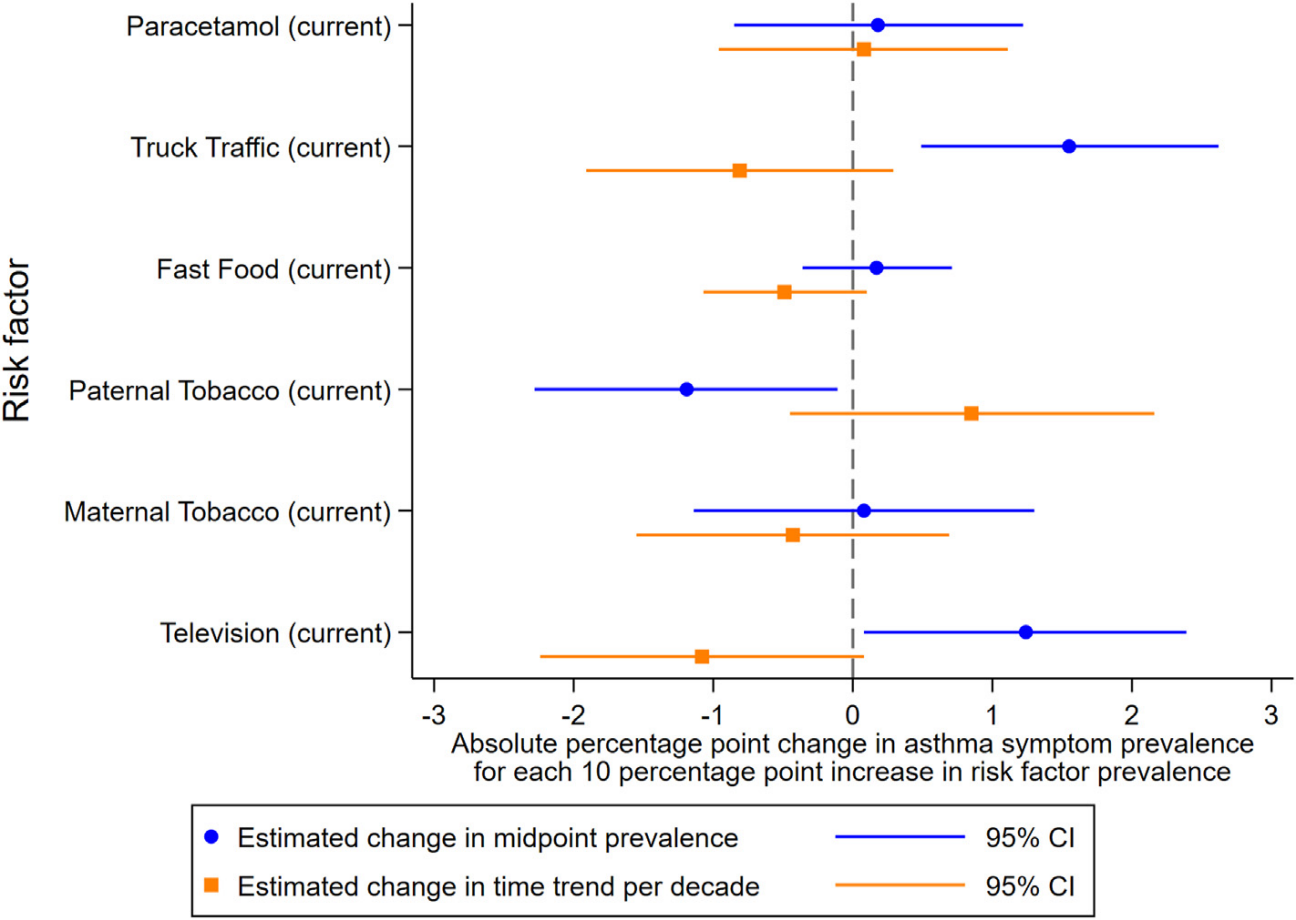
Effects of risk factors from fully adjusted models for adolescents

For adolescents, in the fully adjusted model, it was estimated that decreasing asthma symptom prevalence occurred with regular fast-food consumption (−0.49% points per decade; 95% CI = −1.07, 0.10) and frequent television viewing (−1.08; −2.24, 0.08), but these CIs included the null value, and there was little or no evidence of any other associations between the risk factors and changes in asthma symptom prevalence. Higher mid-point asthma symptom prevalence was associated with truck traffic (1.55% points; 0.49, 2.62) and weakly associated with television viewing (1.24; 0.08, 2.39). Conversely, paternal smoking was associated with a lower mid-point asthma symptom prevalence (−1.19; −2.28, −0.11) (Fig. 4 and Supplemental Table S4).

**Fig. 5:**
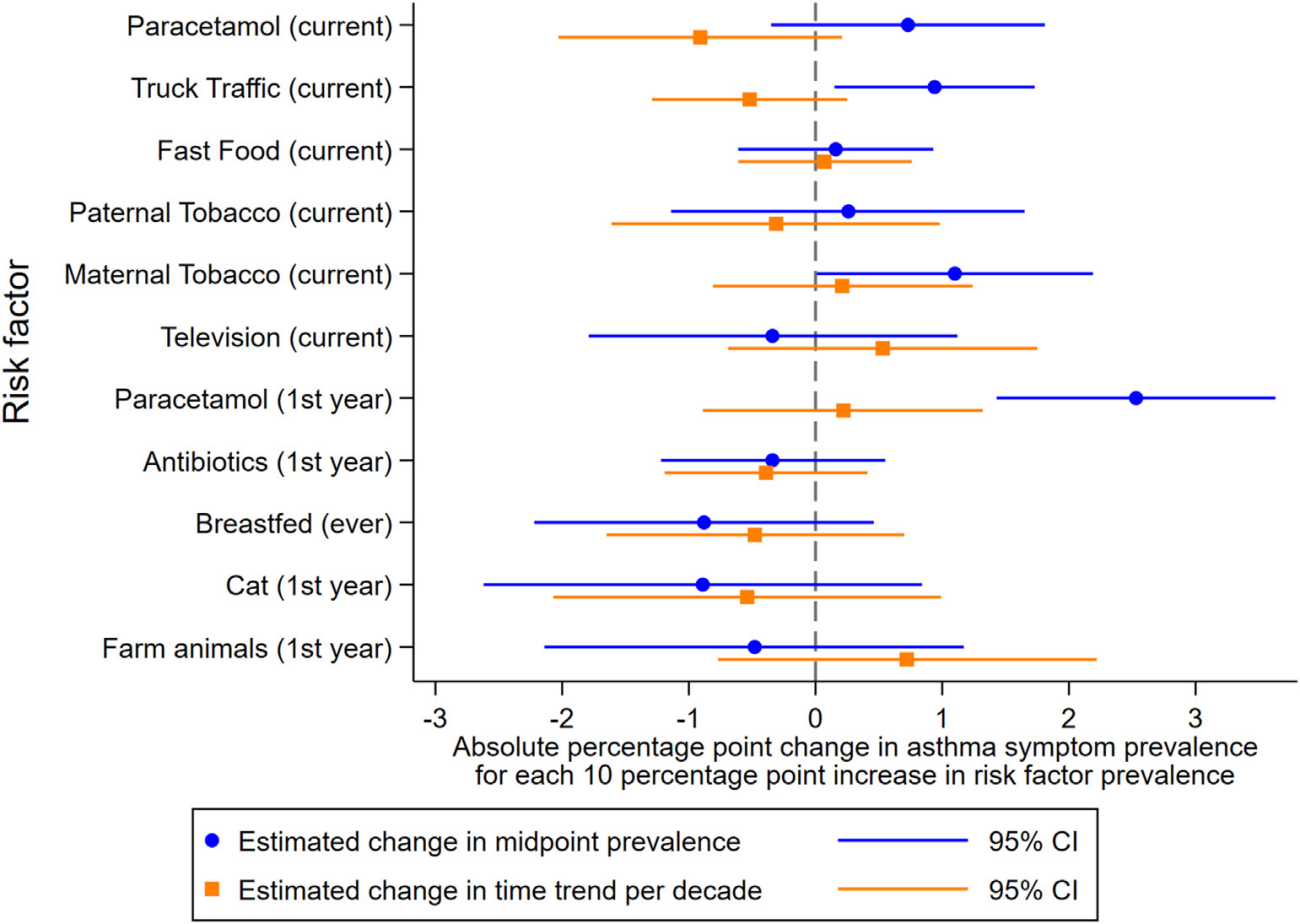
Effects of risk factors from fully adjusted models for children

For children, in the fully adjusted model there was no evidence that any risk factors were associated with trends in asthma symptom prevalence, though there was evidence that truck traffic (0.94; 0.15, 1.73) and paracetamol in the first year (2.53; 1.43, 3.63) were associated with higher asthma symptom prevalence at the mid-point, along with weak evidence for maternal smoking (1.10; 0.01, 2.19) (Fig. 5 and Supplemental Table S5).

In the partially adjusted model for current risk factors, both current paracetamol (−0.94; −1.82, −0.06) and truck traffic (−0.72; −1.41, −0.04) were associated with decreasing asthma symptom prevalence. In the partially adjusted model for early-life risk factors only, antibiotics were associated with decreasing prevalence of asthma symptoms (−0.65; −1.25, −0.05) and paracetamol was associated with higher asthma symptom prevalence at the mid-point (1.55; 0.46, 2.64) (Supplemental Table S5).

### Prediction of current prevalence and time trends: income and region-based results (no individual risk factors)

These models were based on the 121 adolescent and 76 child time trends centres (total 124 different centres shown in Supplemental Fig. S4). The observed time trends in prevalence for these centres can be seen in Supplemental Figs. S5 and S6. The overall predicted values of current asthma symptom prevalence as of January 1, 2019 differed slightly, depending on the model used. When restricting the effects of income/region to be the same across both age groups, the model stratified by income group showed an overall prevalence estimate of 12.8% (95% CI = 11.4%, 14.2%), and the model stratifying by region showed 13.2% (11.9%, 14.6%) (Table 1).

**Table 1:** Estimated 2019 asthma symptom prevalence and time trend, from prediction method with mixed-effect models with random intercepts at country and centre levels, and interactions between time trend, age and country income group, and time trend, age and region

Summary level of predictions		Number of centres in model	Number of centres predicted	Model with 2-way interactions between time and each of the strata^a^		Model with a 3-way interaction between time and the two strata^a^	
				Percentage point change per decade (95% CI)	2019 prevalence (95% CI)	Percentage point change per decade (95% CI)	2019 prevalence (95% CI)
Models with age and country income group strata^a^							
Overall		124	124	−0.06 (−0.79, 0.67)	12.82 (11.40, 14.24)	−0.05 (−0.88, 0.77)	12.80 (11.14, 14.46)
6–7 years		76	124	0.09 (−0.82, 1.00)	12.75 (11.01, 14.49)	0.10 (−1.10, 1.30)	12.70 (10.21, 15.20)
13–14 years		121	124	−0.21 (−1.16, 0.74)	12.89 (11.02, 14.75)	−0.20 (−1.23, 0.82)	12.90 (10.87, 14.92)
6–7 years	Low-income	14	22	−1.37 (−2.86, 0.12)	5.15 (2.54, 7.76)	−1.55 (−3.54, 0.43)	4.85 (1.57, 8.14)
	Lower-middle-income	7	19	1.99 (−0.50, 4.49)	14.25 (9.27, 19.23)	2.00 (−3.72, 7.73)	13.47 (0.83, 26.10)
	Upper-middle-income	21	30	0.50 (−1.21, 2.21)	14.83 (11.63, 18.04)	0.09 (−1.88, 2.06)	15.10 (11.39, 18.80)
	High-income	34	53	−0.22 (−1.39, 0.95)	14.18 (11.83, 16.53)	0.10 (−1.24, 1.45)	14.34 (11.60, 17.08)
13–14 years	Low-income	21	22	−1.67 (−3.07, −0.27)	5.29 (2.81, 7.77)	−1.54 (−3.15, 0.07)	5.50 (2.59, 8.41)
	Lower-middle-income	19	19	1.69 (−0.92, 4.30)	14.39 (9.15, 19.62)	1.69 (−2.04, 5.43)	14.58 (7.18, 21.98)
	Upper-middle-income	30	30	0.19 (−1.49, 1.88)	14.97 (11.83, 18.11)	0.55 (−1.36, 2.46)	15.07 (11.45, 18.69)
	High-income	51	53	−0.52 (−1.75, 0.70)	14.32 (11.85, 16.79)	−0.76 (−2.28, 0.77)	14.13 (11.02, 17.24)
Models with age and region strata^a^							
Overall		124	124	0.16 (−0.52, 0.83)	13.24 (11.94, 14.55)	0.11 (−1.44, 1.67)	13.10 (9.72, 16.49)
6–7 years		76	124	0.42 (−0.47, 1.31)	13.38 (11.66, 15.10)	0.23 (−2.72, 3.18)	12.96 (6.47, 19.45)
13–14 years		121	124	−0.11 (−0.97, 0.76)	13.10 (11.42, 14.78)	0.00 (−0.90, 0.89)	13.25 (11.49, 15.01)
6–7 years	Africa and Eastern Mediterranean	5	16	2.61 (0.04, 5.18)	15.06 (9.99, 20.13)	2.83 (−19.24, 24.89)	12.80 (−36.14, 61.75)^b^
	America	14	25	0.01 (−1.75, 1.77)	16.30 (13.18, 19.42)	−1.10 (−3.01, 0.81)	16.29 (12.90, 19.67)
	Europe	31	50	1.08 (−0.06, 2.22)	15.05 (12.76, 17.33)	0.92 (−0.28, 2.12)	14.46 (12.02, 16.89)
	South-East Asia and Western Pacific	26	33	−1.35 (−2.58, −0.11)	7.83 (5.46, 10.21)	−1.07 (−2.59, 0.46)	8.25 (5.35, 11.15)
13–14 years	Africa and Eastern Mediterranean	16	16	2.09 (−0.43, 4.60)	14.78 (9.80, 19.76)	1.86 (−1.17, 4.88)	14.84 (8.83, 20.86)
	America	24	25	−0.51 (−2.29, 1.27)	16.02 (12.90, 19.14)	0.31 (−1.74, 2.36)	16.61 (12.96, 20.27)
	Europe	49	50	0.56 (−0.60, 1.71)	14.77 (12.40, 17.13)	0.65 (−0.85, 2.14)	15.09 (12.03, 18.14)
	South-East Asia and Western Pacific	32	33	−1.87 (−3.04, −0.69)	7.56 (5.32, 9.79)	−2.12 (−3.42, −0.83)	7.15 (4.65, 9.64)

a All models adjusted for age, country income group and region.

b Confidence intervals outside the bounds of 0–100% points for prevalence. This is due to the linear representation of the outcome; CI=Confidence interval calculated using bootstrapping with 10,000 replicates.

The prevalence of asthma symptoms in low-income countries (age 6–7: 5.2%; 95% CI = 2.5%, 7.8%; age 13–14: 5.3%; 2.8%, 7.8%) was lower than that in lower-middle-, upper-middle- and high-income countries. There was no evidence of a difference between the latter three groups, where estimated prevalence was 14–15% in both age groups. There was evidence that centres in the South-East Asia and Western Pacific regions had lower current prevalence of asthma symptoms (age 6–7: 7.8%; 5.5%, 10.2%; age 13–14: 7.6%; 5.3%, 9.8%) than centres in The Americas and Europe in both age groups, and lower than in Africa and Eastern Mediterranean region in 13-14-year-olds. The highest estimated regional prevalence was for The Americas (age 6–7: 16.3%; 13.2%, 19.4%; age 13–14: 16.0%; 12.9%, 19.1%), although this was not much higher than the estimated prevalence in Europe or the Africa and Eastern Mediterranean region (Table 1).

When the effect of income/region was allowed to vary by age group (three-way interaction models) then the point estimates were not substantially different to those from the two-way interaction models (Table 1). However, the CIs were considerably wider, particularly in the younger age group, reflecting a lack of power to detect the three-way interaction. Despite this low precision, likelihood ratio tests showed that the three-way interaction model was a better fit for the region stratified version (p = 0.004), although not for the income stratified (p = 0.17).

### Prediction: income, region, and risk factor-based results

The addition of risk factors to the model reduced the sample size and the age groups were separated. The adolescent dataset was made up of 50 centres (Fig. 2) and the child dataset 41 centres (Fig. 3).

For adolescents, the findings from the smaller dataset (50 time-trends centres with all risk factors), without including risk factors in the model (Table 2), gave estimates of 2019 prevalence that were consistent with the previous model on the full data set (Table 1). For the trends, there were some differences. High-income countries showed evidence of increasing prevalence (1.7%; 95% CI 0.3%, 3.1%), whereas the analysis on the original dataset showed little evidence of this (despite a small positive point estimate). When risk factors were included in the model, there was very little change to predicted prevalence and predicted trends, but the CIs were wider (Table 2). In both models, with and without risk factors, the CI lower bound for the low-income group was below zero (an impossible value) and the CI was very wide, due to the small number of centres in this group.

**Table 2:** Estimated 2019 asthma symptom prevalence and trend, for age 13–14, from prediction method with mixed-effect models with random intercepts for country and centre. Centres with time trends and risk factor data available, with and without risk factors included (n = 108)

Strata for predictions	Number of centres in model	Without risk factors		With risk factors^a^ (adjusted for and as interaction with time trend)	
		Percentage point change per decade (95% CI)	2019 prevalence % (95% CI)	Percentage point change per decade (95% CI)	2019 prevalence % (95% CI)
Models with interaction between country income group and time, also adjusted for region					
Overall	50	1.06 (−0.19, 2.30)	12.34 (10.08, 14.59)	1.19 (−0.32, 2.71)	12.60 (9.73, 15.47)
Low-income	10	−1.48 (−3.50, 0.54)	2.92 (−0.48, 6.32)^b^	−1.63 (−3.74, 0.48)	2.78 (−1.26, 6.82)^b^
Lower-middle-income	10	3.09 (−0.81, 6.98)	17.58 (9.98, 25.17)	3.52 (−1.06, 8.10)	18.37 (9.28, 27.46)
Upper-middle-income	15	0.75 (−1.82, 3.32)	13.12 (8.86, 17.39)	1.07 (−1.79, 3.93)	13.68 (8.85, 18.52)
High-income	15	1.70 (0.29, 3.11)	14.33 (11.49, 17.17)	1.65 (−0.12, 3.43)	14.22 (10.67, 17.76)
Models with interaction between region and time, also adjusted for country income group					
Overall	50	0.70 (−0.57, 1.97)	11.63 (9.30, 13.95)	0.98 (−0.77, 2.73)	12.15 (8.93, 15.37)
Africa and Eastern Mediterranean	8	2.54 (−1.62, 6.70)	16.31 (7.78, 24.85)	3.05 (−0.82, 6.93)	17.09 (9.50, 24.67)
America	9	0.26 (−3.35, 3.87)	16.15 (10.96, 21.34)	0.56 (−4.26, 5.38)	16.40 (9.15, 23.65)
Europe	16	2.05 (0.46, 3.64)	14.64 (11.40, 17.87)	2.32 (−0.50, 5.13)	15.19 (9.94, 20.43)
South-East Asia and Western Pacific	17	−1.21 (−3.23, 0.81)	4.19 (0.51, 7.87)	−1.04 (−3.25, 1.17)	4.71 (0.45, 8.97)

a Current paracetamol, truck traffic, fast food, maternal smoking, paternal smoking, and television viewing.

b Confidence intervals outside the bounds of 0–100% points for prevalence. This is due to the linear representation of the outcome; CI=Confidence interval calculated using bootstrapping with 10,000 replicates.

For children, the model without risk factors (but on the smaller 41 centre dataset) (Table 3) yielded estimates of 2019 prevalence that were consistent with the model on the larger dataset (Table 1). However, the time trend results were different to those in the previous larger dataset and all groups showed little evidence of any trend. When risk factors were included in the model, there was very little change to predicted prevalence and time trends. The CIs, for both models with and without risk factors, were relatively wide; particularly for the Africa and Eastern Mediterranean region, and both the low- and lower-middle income groups, where they were exceptionally wide due to low numbers in the respective strata. Although the point estimates looked reasonable some of the CI lower bounds were negative (theoretically not possible for a prevalence estimate). This was also due to the wide confidence intervals resulting from a paucity of data in these subgroups (Table 3).

**Table 3:** Estimated 2019 asthma symptom prevalence and trend, for age 6–7, from prediction method with mixed-effect models with random intercepts for country and centre. Centres with time trends and risk factor data available, with and without risk factors included (n = 88)

Strata for predictions	Number of centres	Without risk factors		With early-life risk factors^a^ (adjustment and interaction with time trend)		With current risk factors^b^ (adjustment and interaction with time trend)	
		Percentage point change per decade (95% CI)	2019 prevalence (95% CI)	Percentage point change per decade (95% CI)	2019 prevalence (95% CI)	Percentage point change per decade (95% CI)	2019 prevalence (95% CI)
Models with interaction between country income group and time, also adjusted for region							
Overall	41	0.80 (−1.67, 3.28)	11.83 (7.01, 16.66)	0.88 (−0.67, 2.42)	11.91 (8.90, 14.93)	0.71 (−2.42, 3.84)	11.65 (5.61, 17.69)
Low-income	7	−0.98 (−3.17, 1.20)	2.01 (−2.74, 6.77)^c^	−0.51 (−2.66, 1.65)	3.09 (−1.03, 7.20)^c^	−1.07 (−6.49, 4.35)	1.78 (−10.09, 13.65)^c^
Lower-middle-income	5	3.07 (−15.95, 22.09)	14.23 (−23.16, 51.62)^c^	3.77 (−8.34, 15.88)	15.57 (−8.24, 39.38)^c^	2.32 (−19.93, 24.57)	12.61 (−29.85, 55.06)^c^
Upper-middle-income	13	1.45 (−0.26, 3.15)	12.45 (9.27, 15.62)	1.30 (−0.89, 3.49)	12.05 (8.02, 16.09)	1.60 (−1.09, 4.29)	12.90 (7.77, 18.03)
High-income	16	0.35 (−1.13, 1.83)	14.88 (12.08, 17.68)	0.23 (−1.36, 1.83)	14.52 (11.40, 17.63)	0.26 (−1.69, 2.21)	14.66 (10.80, 18.52)
Models with interaction between region and time, also adjusted for country income group							
Overall	41	1.03 (−4.09, 6.14)	12.22 (0.38, 24.06)	0.83 (−5.32, 6.98)	11.77 (−2.50, 26.04)	0.98 (−1.56, 3.51)	12.10 (7.15, 17.05)
Africa and Eastern Mediterranean	5	4.10 (−37.29, 45.49)	14.95 (−81.43, 111.34)^c^	4.62 (−45.08, 54.32)	16.07 (−99.87, 132.00)^c^	3.64 (−14.56, 21.85)	13.83 (−22.15, 49.80)^c^
America	7	1.79 (−0.96, 4.55)	15.03 (10.51, 19.55)	1.64 (−1.69, 4.96)	14.66 (9.29, 20.03)	1.41 (−2.85, 5.66)	14.21 (7.11, 21.32)
Europe	13	1.11 (−0.26, 2.49)	12.38 (9.69, 15.07)	1.04 (−0.84, 2.93)	12.17 (8.44, 15.91)	1.59 (−0.33, 3.52)	13.41 (9.58, 17.23)
South-East Asia and Western Pacific	16	−0.34 (−1.77, 1.09)	10.01 (7.25, 12.76)	−0.89 (−2.68, 0.91)	8.83 (5.25, 12.42)	−0.54 (−2.13, 1.04)	9.56 (6.46, 12.67)

a Early-life factors (in first year): paracetamol, antibiotics, breastfed ever, cat contact, farm animal contact.

b Current risk factors (in past 12 months): paracetamol, truck traffic, fast food, maternal smoking, paternal smoking, and television viewing.

c Confidence intervals outside the bounds of 0–100% points for prevalence. This is due to the linear representation of the outcome; CI=Confidence interval calculated using bootstrapping with 10,000 replicates.

## Discussion

After adjusting for all available risk factors, along with country income group and region, there were no clear associations between any of the risk factors and changes in asthma prevalence in adolescents. High truck traffic and low paternal smoking rates were associated with high asthma prevalence at the mid-point of the studies in adolescents. Previous individual-level analyses of the ISAAC Phase III data have found positive associations between both maternal and paternal smoking and asthma symptoms.^13^ Thus our current analyses indicate that although maternal and paternal smoking are risk factors for asthma at the individual level, rates of maternal and paternal smoking do not explain global asthma prevalence patterns, and that in fact paternal smoking has a weak negative association with asthma prevalence at the population level.

That regular fast food, frequent television viewing, and paternal smoking showed negative trends with asthma symptom prevalence does not mean they are important preventative factors for asthma. These were weak effects, and there is likely to be residual confounding. For children, where early life factors were available, a number of risk factors were individually associated with a decreasing trend in asthma prevalence, but these associations largely disappeared in the fully adjusted model. However, there was a consistent association between paracetamol use in the first year and a higher mid-point prevalence of asthma. Overall, there were no clear patterns of any associations with the trend in asthma prevalence across age group, or across models with different adjustments.

We can compare these findings to previously published findings at the individual- and school-level.^21^ Although the estimates are not directly comparable due to some being odds ratios and others linear regression coefficients, the general level of evidence of an effect, as well as the magnitude compared to other risk factors, can be assessed. The strongest effects were found at the individual-level, where all risk factors except TV viewing and breastfeeding showed associations with asthma symptoms.^21^ School-level results were more variable, and generally there was lower precision (with wider CIs).^21^ The most important risk factor for children at the centre-level was paracetamol use in the first year of life, which showed a strong effect on centre-level asthma symptom prevalence, although no effect on the time trend of asthma symptom prevalence. The other risk factors with some evidence of association with centre-level prevalence, truck traffic and TV viewing for adolescents and truck traffic and maternal smoking for children, were not associated with time trends in asthma symptom prevalence. All other risk factors showed no effects at the centre-level. This shows that risk factors that were associated with asthma symptoms at the individual level, do not explain the population time trends seen in these studies, and do not explain much of the variation in prevalence at a given time point. This of course relates to overall patterns across centres, it may be possible that in individual centres some risk factors are of more importance than others.

In order for a causal risk factor with a strong association at the individual level to affect prevalence or time trends at the centre-level there are several other criteria that must be met. Firstly, the prevalence of that risk factor must vary significantly between centres.^29^ Risk factors with very little variation in prevalence between centres were excluded from this analysis but the amount of variation required to notice an effect may be substantial. Secondly, the prevalence of asthma symptoms must vary substantially between centres, which does seem to be met. Thirdly, there must not be unmeasured confounders that mask the association at the centre-level, which almost certainly could be an issue as there is considerable unexplained variation at the centre-level which could likely be explained by other unknown factors (and is unlikely to all be due to random fluctuations). Thus, our findings may show that these risk factors do not affect the prevalence of asthma, but only which individuals are affected, or they may individually have only a small effect that is unable to be detected with our sample size.

The extended model provided full predictions, for all centres that had taken part in more than one ISAAC or GAN Phase I, of 2019 prevalence of asthma symptoms along with estimated trends per decade over 27 years. The first model without individual level risk factors estimated the prevalence (predicting in both age groups for each centre) as 12.8% (95% CI 11.4%, 14.2%) if using income group as the main predictor and 13.2% (11.9%, 14.6%) if using region. Low-income countries were predicted to have lower prevalence of asthma symptoms, with evidence of a decreasing trend in adolescents. Centres in the South-East Asia and Western Pacific region were predicted to have lower prevalence than other regions, and that their prevalence was also decreasing. This finding is unexpected, since it has been hypothesized that prevalence would increase in these countries with increasing westernisation.^30^

We note that there was still substantial heterogeneity between centres, within strata, that may be explained by other risk factors or possible external events eg, Syria showed a very large increase in asthma symptoms following an extended period of civil war.

Despite these limitations, the method of predicting prevalence at one time-point appeared to work well.

### Strengths and limitations of the study data

The main strength of the ISAAC and GAN surveys was the standardised methodology used around the world across three phases of data collection spanning 27 years. Standardised questionnaires (translated and back translated) included descriptions of symptoms, which are less affected by healthcare practices and differences in diagnoses. The large number of participants and high response rate within the studies was also a strength, providing the power to identify risk factors with smaller effects. However, this benefit was diluted when moving to a centre prevalence-level dataset for time trends analysis.

A limitation was the number of centres that took part in GAN Phase I. There were a lot of centres that expressed interest, but not all were able to conduct the surveys. This limited the overlap of centres between ISAAC and GAN, and therefore the data available for analysing time trends; in particular, there were not enough data to consider changes in risk factors over time (with risk factors only available for ISAAC Phase III and GAN Phase I). Additionally, the centres were not representative of the world (or the WHO regions), with some countries providing data from multiple centres yet many countries not included at all, making it impossible to produce valid global estimates.

When examining time trends, the centres with only one time point of data were excluded, leading to a smaller sample size and loss of statistical power. It should also be noted that the three time points of surveys involved different individuals (as this was a series of cross-sectional surveys with different participants), and often involved different schools. It may have been useful to use the same schools so that trends could be followed at school-level which would keep more information than centre-level, although on the other hand, this could mean that the surveys would become non-representative since they would not include newly established schools. As it is, all outcomes and risk factors were summarised to centre prevalence-level so there was only one record per centre per time point per age group. For example, paracetamol use may have a substantial effect at the individual level but there would have to be large differences in the prevalence of paracetamol use between different centres for an association to be reflected at the centre-level.

With any cross-sectional survey there are always concerns about bias and the temporality of exposures and outcomes needs to be considered. In the ISAAC and GAN questionnaires, both the outcome and most of the current risk factors were assessed over the preceding 12 months. In the child questionnaire the early life questions were based on the first year of life which would probably be before the onset of asthma.

Another area that might cause bias is misclassification of the exposure(s) and/or the outcome. Most questions in the ISAAC and GAN studies were categorical, but were transformed to binary variables for the analyses. In fully adjusted models, if multiple exposures were affected by misclassification, even when non-differential, effects could be biased in either direction. Similarly, recall bias could be an issue in the study, particularly when answering questions about early life. However, many of the questions were on fairly easy to remember facts which would not be as susceptible to recall bias (eg, pets, breastfeeding, parental smoking, birthweight). Additionally, income data was only available at the country-level. It is possible some centres serve a population with higher/lower than average income for the country, but we have used broad definitions (4 categories) and the areas were not chosen for being specifically rich or poor.

Unmeasured or residual confounding is also likely to be an issue in these analyses. The risk factors were mainly binary, such as maternal smoking, therefore different frequencies of smoking were not captured. There was also considerable unexplained variation in symptom prevalence between centres which implies there was unmeasured confounding from data that was not available in our studies. This is not a surprise, as there is still a great deal that is not known about the causes of asthma.

For the time trends analyses, all models were fitted using an assumption of linearity in the time trend. Although statistically there was no evidence against linearity overall in the models, it is likely that many centres had non-linear changes in prevalence, and there could be different true patterns for different groups. However, given the lack of improvement to the model through the addition of a spline, and the available sample size for the analysis, more complex models of the time trends would have been unlikely to see improvements in fit or predictions. The models would certainly be underpowered to detect different shapes of time trend in different groups.

With risk factor data specific to each time point, we would have liked to model the association between the change in prevalence of risk factors and the change in prevalence of asthma symptoms (centre-level longitudinal with time-varying risk factor covariates). However, in ISAAC Phase I there were no risk factor data, and the overlap between ISAAC Phase III and GAN Phase I centres was quite small (26 centres) which would be underpowered to detect most associations.

## Conclusions

The ISAAC and GAN data sets are large and unique. Using these data sets, the best overall estimate of the current prevalence of asthma symptoms in adolescents and children is 12.8% (95% CI = 11.4%, 14.2%), with no significant difference between age groups, incorporating all 124 centres that took part in more than one ISAAC/GAN survey.

The risk factors that were identified at the individual level do not seem to explain the differences between centres and countries, or the time trends. Income group and region account for some of the differences, but the unexplained variation is still very high. Perhaps the effects of some of these risk factors are too small to be identified in a centre-level analysis, and maybe other types of higher-level risk factors that are more detailed (eg, summary markers of Westernisation^30^) could have more explanatory power.

Global patterns in asthma symptoms are extremely complex with substantial variation in both absolute levels and trends. There is no clear overall change in prevalence, but this conclusion may hide problem areas where the prevalence of asthma is increasing greatly. There is also the concern that even in areas where prevalence is not increasing, the burden of asthma may still be substantial, and asthma management may be poor.

## Abbreviations

ISAAC, The International Study of Asthma and Allergies in Childhood; GAN, Global Asthma Network; BLUPs, Best linear unbiased predictions; CI, Confidence interval; WHO, World Health Organization.

## Funding statement

The ISAAC International Data Centre was supported by the 10.13039/501100001505Health Research Council of New Zealand (NZ), the Asthma and Respiratory Foundation of NZ, the 10.13039/501100002845Child Health Research Foundation NZ, the 10.13039/501100009187Hawke's Bay Medical Research Foundation NZ, the 10.13039/501100001529Waikato Medical Research Foundation NZ, Glaxo Wellcome NZ, the NZ Lottery Board NZ, and Astra Zeneca NZ. Glaxo Wellcome International Medical Affairs supported the regional coordination and the ISAAC International Data Centre.

The GAN Global Centre in Auckland was funded by The 10.13039/501100001537University of Auckland with additional funding from The 10.13039/501100005850International Union Against Tuberculosis and Lung Disease France, Boehringer Ingelheim NZ, and an Astra Zeneca Educational Grant NZ. The London Data Centre was supported by a PhD studentship [to CER] from the UK 10.13039/501100000265Medical Research Council (grant number MR/N013638/1) and funding from the 10.13039/501100000781European Research Council under the European Union's Seventh Framework Programme (FP7/2007–2013, ERC grant agreement number 668954). The Murcia Data Centre was supported by the 10.13039/501100004687University of Murcia and by 10.13039/501100004587Instituto de Salud Carlos III, fund PI17/0170. We thank the NIHR Global Health Research Unit on Lung Health and TB in Africa at 10.13039/100014976Liverpool School of Tropical Medicine – “IMPALA” for helping to make this work possible (grant number 16/136/35); IMPALA was commissioned by the 10.13039/501100000272National Institute for Health Research (NIHR) 10.13039/100006090Global Health Research portfolio using UK aid from the UK Government.

The views expressed in this publication are those of the authors and not necessarily those of any of the funders.

## Availability of data and materials

ISAAC data are already deposited for wider use. The study protocol including a recommended informed consent form and statistical analysis plan are in the public domain. The GAN Phase I data, including de-identified individual participant data, will be made available on the Global Asthma Network website http://www.globalasthmanetwork.org/ within 12 months of all GAN Phase I analyses being published. Access will require a formal request, a written proposal and a signed data access agreement.

## Contributorship

CER completed the analysis and data visualization and RJS, NP and DPS supervised the analysis. CER wrote the original draft and RJS, NP, DPS and the GAN phase I Study Group reviewed and edited the manuscript. In the GAN Phase I Study Group, the Steering Committee (including NP and DPS) conceptualised the study and wrote the protocols, the centre PIs and their colleagues collected the data and the data centres (including CER, RJS and NP) checked, cleaned and managed the data.

## Ethics approval

All centres were required to attain approval from their local ethics committee. They determined the method of consent as either passive (agreeing by participation) or active (signing a written consent prior to filling in the questionnaire) from parents/caregivers; however, GAN recommended passive consent because active (written) consent could reduce the response rate. Because adolescents should also manifest their own consent, they agreed by participating.

## Authors consent for publication

All authors agree to the publication of this manuscript in World Allergy Organization Journal. The authors confirm the manuscript is original, has not been published before (except in the academic thesis of CER) and is not being considered for publication elsewhere.

## Declaration of competing interest

The authors report no competing interests.
