# Supplementary material for: Potential asthma risk factors do not account for global asthma symptom prevalence patterns and time trends in children and adolescents
Source: World Allergy Organ J. 2024 Jun 14;17(6):100917. doi: 10.1016/j.waojou.2024.100917 (PMC11227011; doi:10.1016/j.waojou.2024.100917)
Supplement: Multimedia component 1 [file mmc1.pdf]

Supplemental material

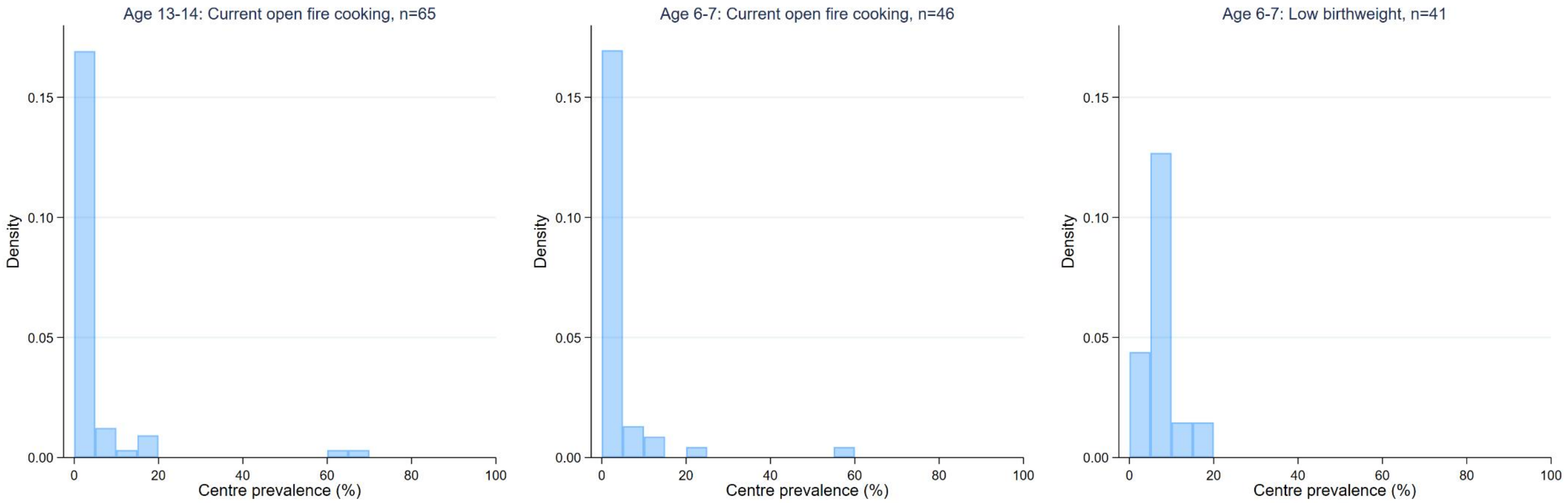

Figure S1 Distributions of risk factors not included in final analysis

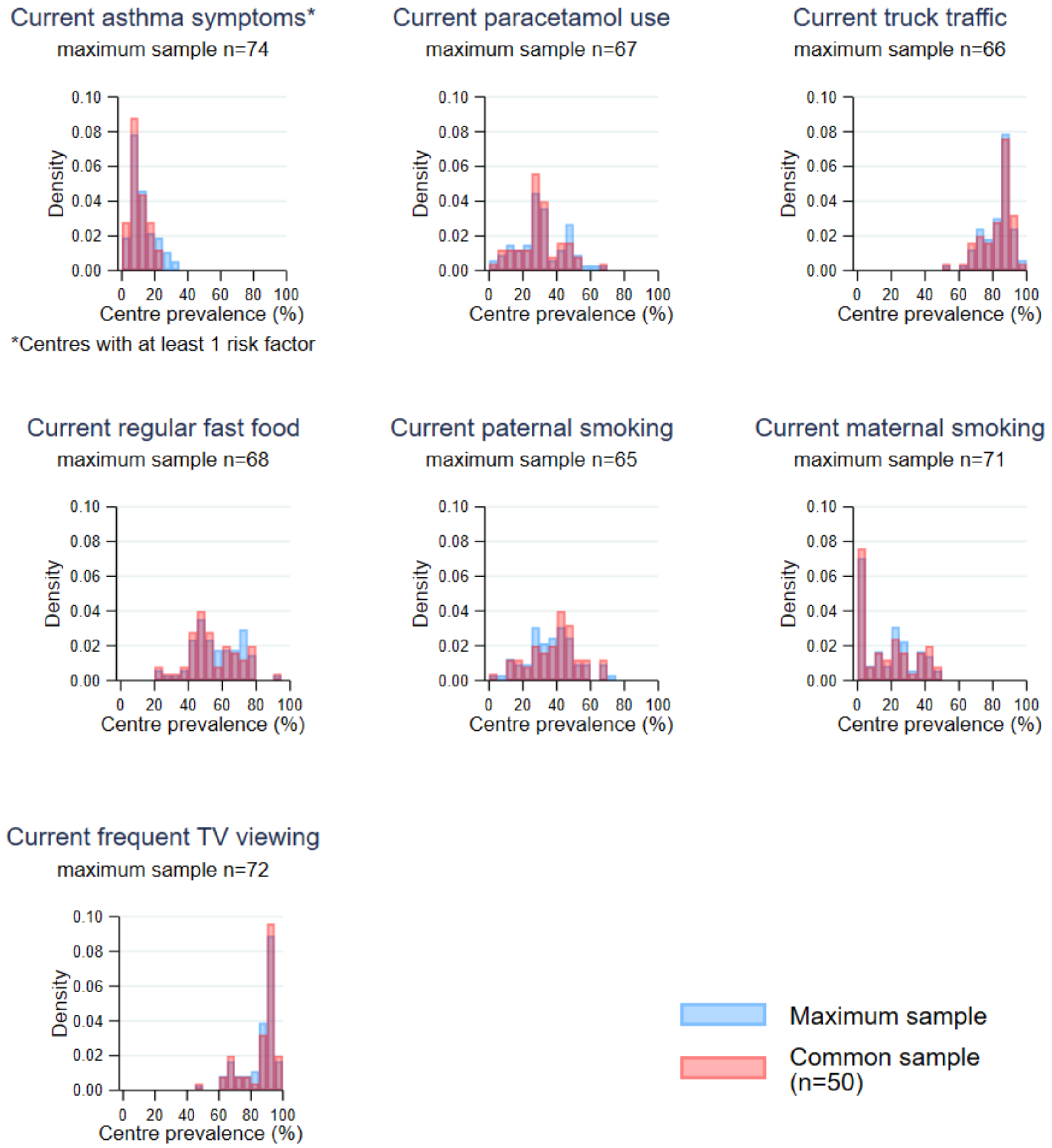

Figure S2 Distributions of risk factors for adolescents aged 13-14 years

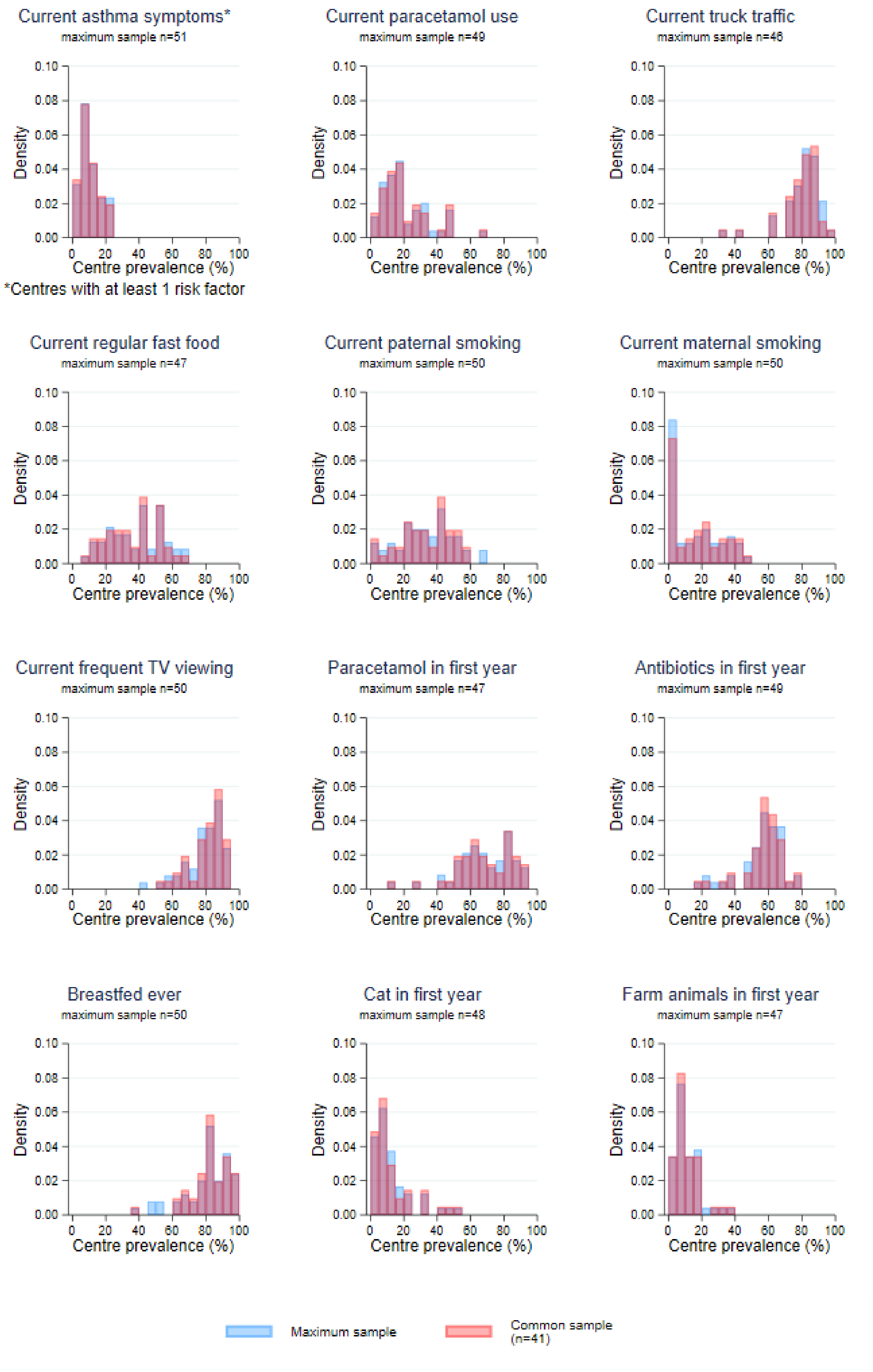

Figure S3 Distributions of risk factors for children aged 6-7 years

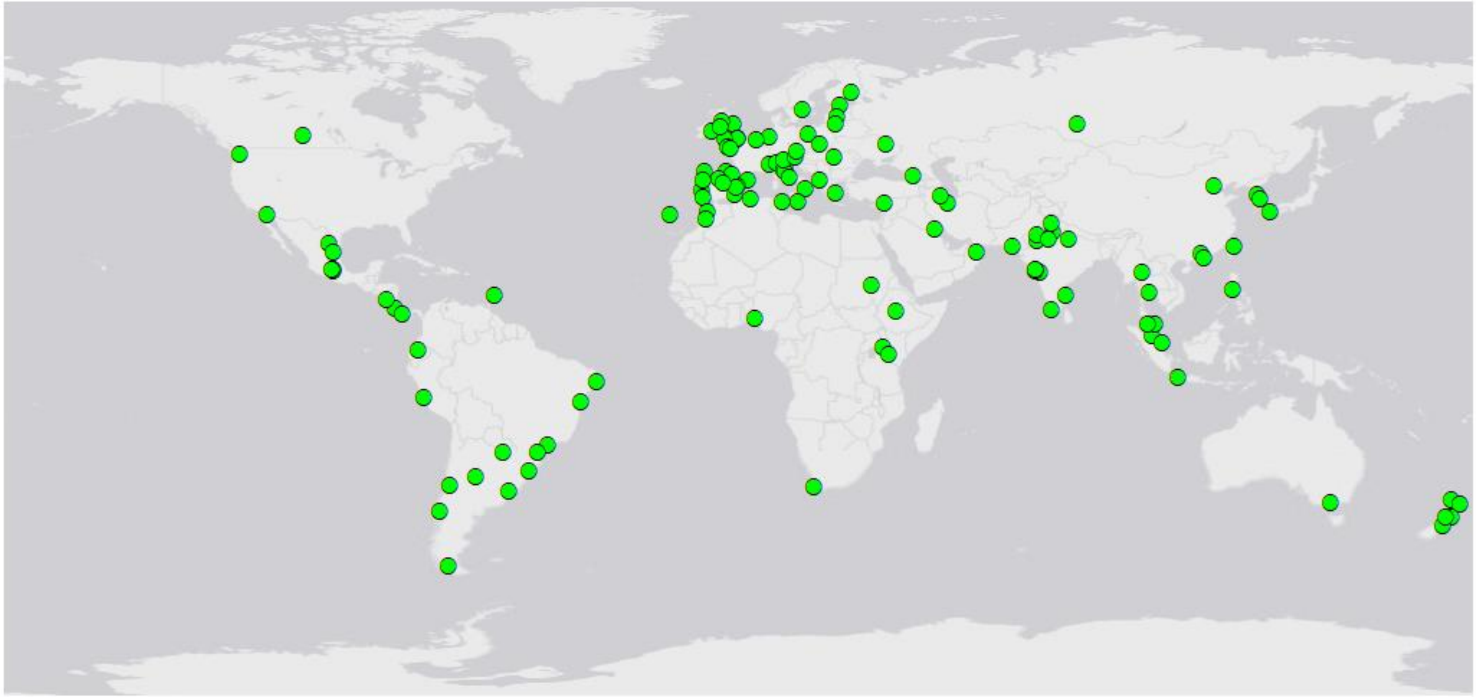

Figure S4 All time trends centres used in the prediction model without risk factors

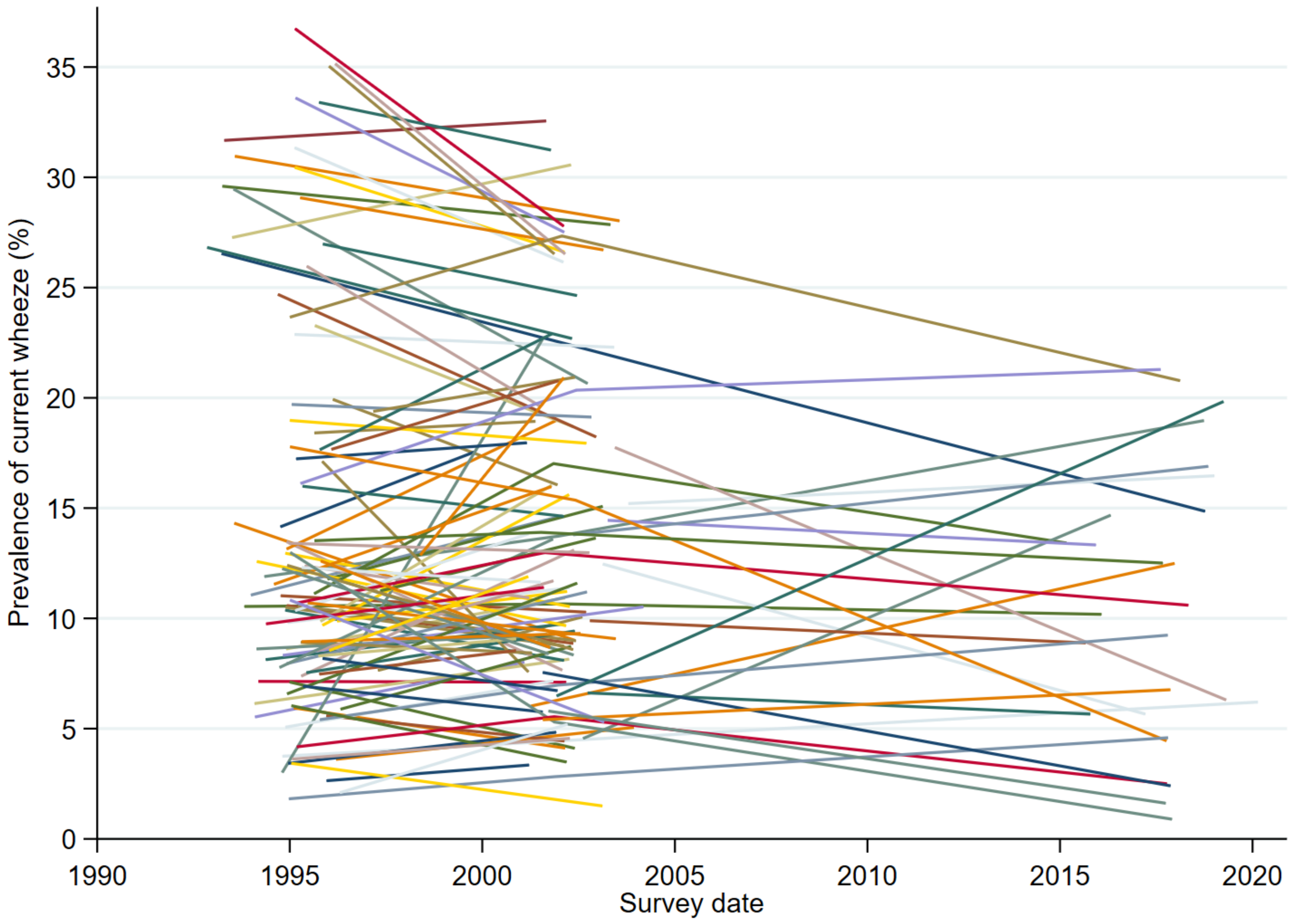

Figure S5 Observed prevalence by centre, adolescents aged 13-14 years

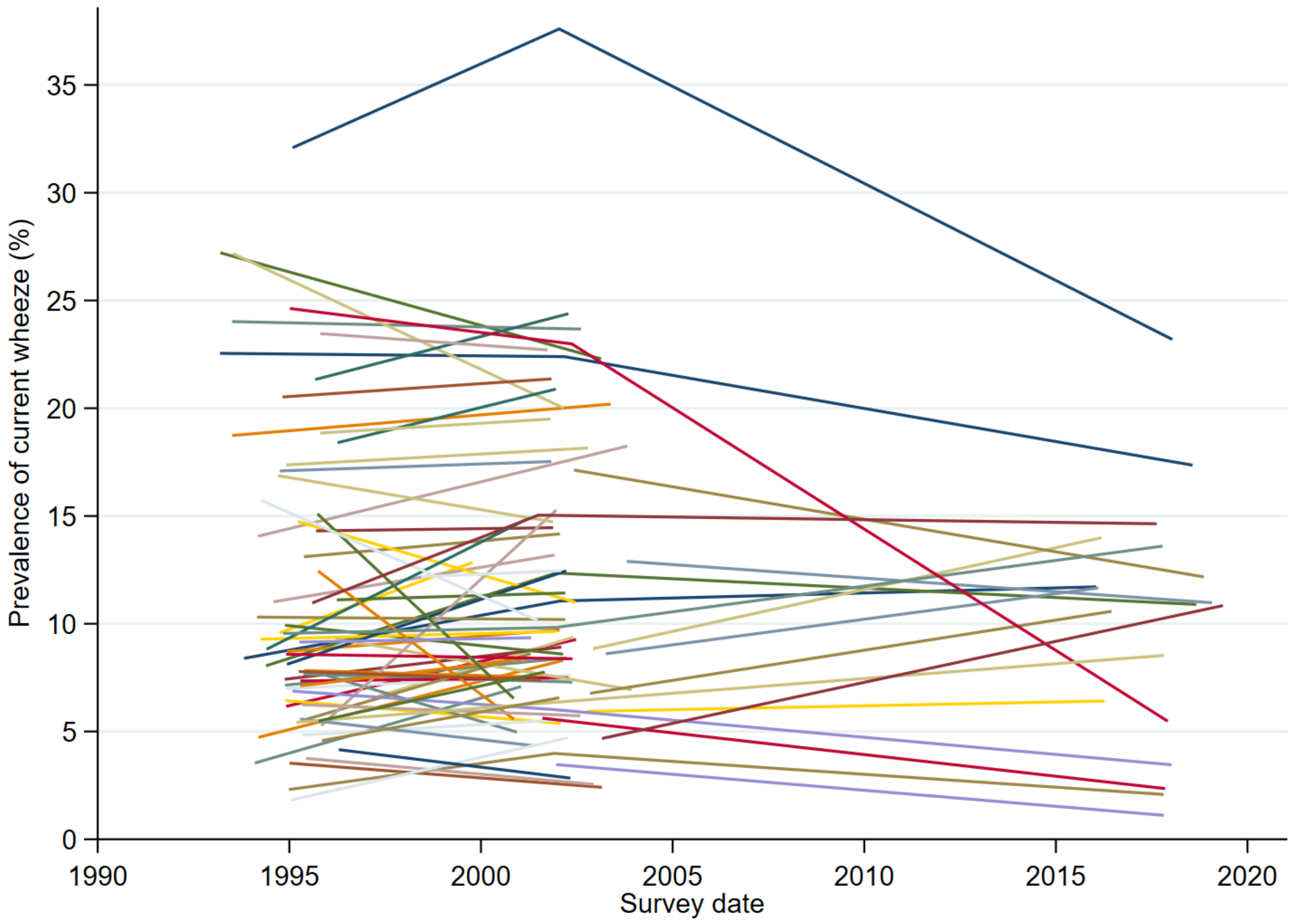

Figure S6 Observed prevalence by centre, children aged 6-7 years

Table S1 Definitions of risk factors

Age group	Risk Factor	Question	Positive Response
6-7 years (question asked to parent)	Paracetamol (current)	In the past 12 months, how often, on average, have you given your child paracetamol?	At least once per month
	Heavy truck traffic (current)	How often do trucks pass through the street where you live, on weekdays?	Frequently or almost the whole day
	Fast food (current)	In the past 12 months, how often, on average did your child eat fast food / burgers?	At least once a week
	Paternal tobacco (current)	Does your child's father (or male guardian) smoke cigarettes?	Yes
	Maternal tobacco (current)	Does your child's mother (or female guardian) smoke cigarettes?	Yes
	Television (current)	During a normal week, how many hours a day (24 hours) does your child watch television?	At least one hour per day
	Open fire cooking (current)	In your house, what fuels are usually used for cooking? Electricity, gas, open fires, other	Any that include open fires
	Low birthweight	What was the weight of your child when he / she was born?	Less than 2.5kg
	Paracetamol (1st year)	In the first 12 months of your child's life, did you usually give paracetamol for fever?	Yes
	Antibiotics (1st year)	In the first 12 months of your child's life, did your child have any antibiotics?	Yes
	Breastfed ever	Was your child breastfed?	Yes
	Cat (1st year)	Did you have a cat in your home during the first year of your child's life?	Yes
	Farm animals (prenatal)	Has the child's mother had regular (at least once a week) contact with farm animals (e.g. cattle, pigs, goats, sheep or poultry) while being pregnant with this child?	Yes
13-14 years (question asked to child)	Paracetamol (current)	In the past 12 months, how often, on average, have you taken paracetamol?	At least once per month
	Heavy truck traffic (current)	How often do trucks pass through the street where you live, on weekdays?	Frequently or almost the whole day
	Fast food (current)	In the past 12 months, how often, on average did you eat fast food / burgers?	At least once a week
	Paternal tobacco (current)	Does your father (or male guardian) smoke cigarettes?	Yes
	Maternal tobacco (current)	Does your mother (or female guardian) smoke cigarettes?	Yes
	Television (current)	During a normal week, how many hours a day (24 hours) do you watch television?	At least one hour per day
	Open fire cooking (current)	In your house, what fuels are usually used for cooking? Electricity, gas, open fires, other	Any that include open fires

Table S2 Details of centres with data for all included risk factors

Grouped region	Country name	Income level	Centre name	Sample size of age 6-7 data			Sample size of age 13-14 data		
				GAN Phase I	ISAAC Phase III	ISAAC Phase I	GAN Phase I	ISAAC Phase III	ISAAC Phase I
Africa and Eastern Mediterranean	Iran	LM	Rasht		3057	3013		3004	3181
Africa and Eastern Mediterranean	Iran	LM	Tehran		3008	2456		3119	2691
Africa and Eastern Mediterranean	Morocco	LM	Casablanca					1777	3183
Africa and Eastern Mediterranean	Morocco	LM	Marrakech					1689	2900
Africa and Eastern Mediterranean	Nigeria	L	Ibadan		2396	1696	2897	3142	3057
Africa and Eastern Mediterranean	Oman	UM	Al-Khod		4130	3891		3747	3174
Africa and Eastern Mediterranean	South Africa	LM	Cape Town				3979	5037	5169
Africa and Eastern Mediterranean	Syrian Arab Republic	LM	Latakia	1116	2373		1215	3010	
America	Barbados	UM	Barbados		2759	3289		2498	3533
America	Brazil	UM	Salvador					3020	3162
America	Canada	H	Saskatoon		1255	2418			
America	Chile	UM	South Santiago		3075	3182	2750	3026	3050
America	México	UM	Ciudad Victoria	2444	2603		2468	3122	
America	México	UM	Mexicali	2001	2568		2479	2988	
America	México	UM	Monterrey				2641	3006	
America	México	UM	México City (North Area)	2515	3205		3375	3891	
America	México	UM	Toluca Urban Area	2712	3235				
America	Peru	LM	Lima					3022	3157
America	Uruguay	UM	Montevideo					3177	3072
Europe	Belgium	H	Antwerp		5645	6533			
Europe	Estonia	UM	Tallinn		2385	3070		3603	3506
Europe	Finland	H	Kuopio County					3051	2876
Europe	Lithuania	UM	Kaunas		2772	1878		2723	1600
Europe	Poland	UM	Krakow (1995)		2497	2264		2545	2786
Europe	Poland	UM	Poznan		1999	2710		1875	3625
Europe	Portugal	H	Funchal		1819	1797		3161	3531
Europe	Portugal	H	Lisbon		2477	2143		3024	3030
Europe	Portugal	H	Portimao		1069	1189		1109	1058
Europe	Portugal	H	Porto					3336	3131
Europe	Spain	H	A Coruña	3407	3016		3462	2979	

Europe	Spain	H	Barcelona					3066	3031
Europe	Spain	H	Bilbao	2707	3157	3019	3379	3401	3211
Europe	Spain	H	Cartagena	3509	2948	3335	3437	3998	3017
Europe	Spain	H	Madrid		2347	2442		2652	3221
Europe	Spain	H	Valencia		3398	3940		3132	3174
Europe	Spain	H	Valladolid					2944	3177
South-East Asia and Western Pacific	China	LM	Beijing					3530	4166
South-East Asia and Western Pacific	China	LM	Guangzhou					3514	3855
South-East Asia and Western Pacific	Hong Kong SAR, China	H	Hong Kong					3321	4666
South-East Asia and Western Pacific	India	L	Bikaner				2702	3059	
South-East Asia and Western Pacific	India	L	Borivali					1004	3878
South-East Asia and Western Pacific	India	L	Chennai (3)					2181	3079
South-East Asia and Western Pacific	India	L	Jodhpur		2114	1104		2341	1080
South-East Asia and Western Pacific	India	L	Lucknow	2969	3000		2969	3000	
South-East Asia and Western Pacific	India	L	Mumbai (16)		2865	3967			
South-East Asia and Western Pacific	India	L	Mumbai (18)		4862	3568		2982	3177
South-East Asia and Western Pacific	India	L	New Delhi (7)	2516	3706	2938	3024	3469	3025
South-East Asia and Western Pacific	India	L	Pune				3030	1983	2696
South-East Asia and Western Pacific	Indonesia	L	Bandung		2503	1371		2826	2247
South-East Asia and Western Pacific	Japan	H	Fukuoka		2958	2896		2520	2827
South-East Asia and Western Pacific	Malaysia	UM	Alor Setar		3786	2978		2941	3298
South-East Asia and Western Pacific	Malaysia	UM	Kota Bharu		3110	3819		2989	3075
South-East Asia and Western Pacific	New Zealand	H	Auckland	1538	3541	3526			
South-East Asia and Western Pacific	New Zealand	H	Bay of Plenty		2150	2681			
South-East Asia and Western Pacific	New Zealand	H	Christchurch		3315	3318			
South-East Asia and Western Pacific	New Zealand	H	Nelson		1867	1868			
South-East Asia and Western Pacific	Philippines	LM	Metro Manilla					3658	3207
South-East Asia and Western Pacific	Taiwan, China	H	Taipei	3036	4832	4806	3474	6378	11003
South-East Asia and Western Pacific	Thailand	LM	Bangkok	3067	4209	3629			
South-East Asia and Western Pacific	Thailand	LM	Chiang Mai		3106	3828			

Table S3 Missing risk factor information in centres with included data for all risk factors

Risk factor	Age 13-14 (50 centres)		Age 6-7 (41 centres)	
	Median % missing	1st Quartile - 3rd Quartile	Median % missing	1st Quartile - 3rd Quartile
Paracetamol (current)	2.5	1.2 - 4.0	3.4	1.9 - 5.1
Heavy truck traffic (current)	1.3	0.6 - 2.3	2.2	1.2 - 3.3
Fast food (current)	2.5	1.4 - 5.1	5.2	2.8 - 7.8
Paternal tobacco (current)	1.7	0.9 - 2.8	2.7	1.7 - 3.8
Maternal tobacco (current)	1.4	0.8 - 2.2	1.4	0.6 - 2.5
Television (current)	1.1	0.6 - 1.8	1.9	1.0 - 2.8
Paracetamol (1st year)			2.7	1.8 - 4.6
Antibiotics (1st year)			4.8	3.3 - 6.5
Breastfed ever			2.1	1.1 - 3.0
Cat (1st year)			1.4	0.6 - 2.4
Farm animals (1st year)			1.7	0.9 - 3.0

Table S4 Risk factor associations with prevalence and time trends of asthma symptoms with random intercepts for centre and country, age 13-14

Risk factor (effect of 10 percentage point higher prevalence)	Minimally adjusted[a] models (n=108)		Fully adjusted[b] model (n=108)	
	Effect on asthma symptom prevalence at 1st Jan 2002 (95% CI)	Effect on change in asthma symptom prevalence per decade (95% CI)	Effect on asthma symptom prevalence at 1st Jan 2002 (95% CI)	Effect on change in asthma symptom prevalence per decade (95% CI)
Current paracetamol	0.20 (-0.91, 1.31)	0.09 (-0.92, 1.10)	0.18 (-0.85, 1.22)	0.08 (-0.96, 1.11)
Current truck traffic	1.52 (0.42, 2.61)	-0.66 (-1.73, 0.41)	1.55 (0.49, 2.62)	-0.81 (-1.91, 0.29)
Current regular fast food	0.23 (-0.33, 0.80)	-0.61 (-1.14, -0.07)	0.17 (-0.36, 0.71)	-0.49 (-1.07, 0.10)
Current paternal smoking	-0.90 (-1.81, 0.02)	0.21 (-0.83, 1.25)	-1.19 (-2.28, -0.11)	0.85 (-0.45, 2.16)
Current maternal smoking	-0.45 (-1.54, 0.64)	-0.01 (-0.91, 0.90)	0.08 (-1.14, 1.30)	-0.43 (-1.55, 0.69)
Current frequent television	0.63 (-0.55, 1.80)	-1.14 (-2.13, -0.14)	1.24 (0.08, 2.39)	-1.08 (-2.24, 0.08)

[a]Minimally adjusted for income group and region. [b]Fully adjusted for income group, region and all other risk factors in the table.

Table S5 Risk factor associations with prevalence and time trends of asthma symptoms with random intercepts for centre and country, age 6-7

Risk factor (effect of 10% absolute increase)	Minimally adjusted[a] models (n=88)		Partially adjusted[b] models (n=88)		Fully adjusted[c] model (n=88)	
	Effect on asthma symptom prevalence at 1st Jan 2002 (95% CI)	Effect on change in asthma symptom prevalence per decade (95% CI)	Effect on asthma symptom prevalence at 1st Jan 2002 (95% CI)	Effect on change in asthma symptom prevalence per decade (95% CI)	Effect on asthma symptom prevalence at 1st Jan 2002 (95% CI)	Effect on change in asthma symptom prevalence per decade (95% CI)
Current paracetamol	1.05 (-0.09, 2.19)	-0.82 (-1.45, -0.19)	0.68 (-0.44, 1.81)	-0.94 (-1.82, -0.06)	0.73 (-0.35, 1.81)	-0.91 (-2.03, 0.21)
Current truck traffic	-0.11 (-1.07, 0.85)	-0.96 (-1.66, -0.26)	0.30 (-0.60, 1.20)	-0.72 (-1.41, -0.04)	0.94 (0.15, 1.73)	-0.52 (-1.29, 0.25)
Current regular fast food	0.50 (-0.15, 1.15)	0.09 (-0.44, 0.62)	0.32 (-0.38, 1.02)	0.33 (-0.22, 0.89)	0.16 (-0.61, 0.93)	0.07 (-0.61, 0.76)
Current paternal smoking	-0.23 (-1.48, 1.02)	0.51 (-0.21, 1.22)	-1.01 (-2.33, 0.31)	0.01 (-0.80, 0.82)	0.26 (-1.14, 1.65)	-0.31 (-1.61, 0.98)
Current maternal smoking	0.40 (-0.84, 1.63)	-0.90 (-1.64, -0.16)	0.79 (-0.57, 2.14)	-0.36 (-1.18, 0.47)	1.10 (0.01, 2.19)	0.21 (-0.81, 1.24)
Current frequent television	0.60 (-0.75, 1.95)	-0.35 (-1.20, 0.49)	0.58 (-0.92, 2.08)	0.19 (-0.76, 1.13)	-0.34 (-1.79, 1.12)	0.53 (-0.69, 1.75)
Paracetamol in first year	1.27 (0.46, 2.08)	-0.40 (-0.69, -0.11)	1.55 (0.46, 2.64)	0.17 (-0.51, 0.85)	2.53 (1.43, 3.63)	0.22 (-0.89, 1.32)
Antibiotics in first year	0.23 (-0.47, 0.93)	-0.84 (-1.24, -0.45)	0.06 (-0.66, 0.79)	-0.65 (-1.25, -0.05)	-0.34 (-1.22, 0.55)	-0.39 (-1.19, 0.41)
Breastfed ever	0.30 (-1.06, 1.66)	-0.82 (-1.30, -0.34)	-0.21 (-1.64, 1.22)	-0.34 (-1.38, 0.71)	-0.88 (-2.22, 0.46)	-0.48 (-1.65, 0.70)
Cat in first year	0.31 (-1.00, 1.63)	-0.74 (-1.31, -0.17)	-0.51 (-2.24, 1.23)	-0.41 (-1.74, 0.93)	-0.89 (-2.62, 0.84)	-0.54 (-2.07, 0.99)
Farm animals in first year	-0.47 (-1.97, 1.03)	-0.45 (-1.58, 0.68)	-0.56 (-2.14, 1.03)	0.47 (-0.76, 1.69)	-0.48 (-2.14, 1.17)	0.72 (-0.77, 2.22)

[a]Minimally adjusted for income group and region. [b]Partially adjusted for all risk factors on the same side of the thick line. [c]Fully adjusted for income group, region and all other risk factors in the table.
